# Fast-TIPL Occurs for Salient Images without a Memorization Requirement in Men but Not in Women

**DOI:** 10.1371/journal.pone.0036228

**Published:** 2012-04-27

**Authors:** Virginie Leclercq, Aaron R. Seitz

**Affiliations:** Department of Psychology, University of California Riverside, Riverside, California, United States of America; National Institute of Mental Health, United States of America

## Abstract

Recent research of task-irrelevant perceptual learning (TIPL) demonstrates that stimuli that are consistently presented at relevant point in times (e.g. with task-targets or rewards) are learned, even in the absence of attention to these stimuli. However, different research paradigms have observed different results for how salient stimuli are learned; with some studies showing no learning, some studies showing positive learning and others showing negative learning effects. In this paper we focused on how the level of processing of stimuli impacts fast-TIPL. We conducted three different experiments in which the level of processing of the information paired with a target was manipulated. Our results indicated that fast-TIPL occurs when participants have to memorize the information presented with the target, but also when they just have to process this information for a secondary task without an explicit memorization of those stimuli. However, fast-TIPL does not occur when participants have to ignore the target-paired information. This observation is consistent with recent models of TIPL that suggest that attentional signals can either enhance or suppress learning depending on whether those stimuli are distracting or not to the subjects' objectives. Our results also revealed a robust gender effect in fast-TIPL, where male subjects consistently show fast-TIPL, whereas the observation of fast-TIPL is inconsistent in female subjects.

## Introduction

Our memory is selective. We remember some information that we know is important and other information seems to just pop-into our head despite how unimportant it might seem to us. What are the rules that determine what we remember? It is tempting to believe that learning and memory are primarily guided by conscious processes, but there is evidence that implicit factors play key roles in determining what information we encode [1], [2].

Recently, a number of studies of task-irrelevant perceptual learning (TIPL) demonstrated that processing the target of a rapid serial visual presentation (RSVP) detection task can facilitate encoding of irrelevant, and even unnoticed, information paired with these targets [3]–[6]. More precisely, for example, when subjects are exposed to a motion direction stimulus while performing a RSVP task, it was found that after many days of exposure to this procedure, the subjects became better at discriminating and detecting the exposed motion direction paired with the target of the RSVP task even if the motion stimulus was presented at a subliminal level and was irrelevant for the central task of the subjects. However, while initial accounts of TIPL had the goal of establishing that reinforcement in the absence of attention could lead to TIPL [3], [6], [7], [8], recent accounts of TIPL discuss a more complex interplay between attention and reinforcement whereby attentional signals guide learning by suppressing distracting features while permitting the learning of important features [5], [9], [10], [11]. Indeed, TIPL has been observed in some studies but not in others and the role of attention in TIPL can explain this discrepancy in results. More precisely, TIPL has been observed in studies where the information paired with the target was parathreshold, but not in studies where the information paired with the target was supra-threshold [11], [12]. One hypothesis is that weak task-irrelevant signals fail to be “noticed”, and to be suppressed by the attentional system and thus are learned, while stronger stimulus signals are detected, suppressed, and are not learned [9], [10].

Another version of experiments of TIPL (fast-TIPL) used supra-threshold stimuli (instead of sub-threshold stimuli) as irrelevant information presented with the stream containing the target and allows studying TIPL within one trial (instead of thousands of trials necessary in the classic paradigm of TIPL, [7]). In the fast-TIPL paradigm, subjects conducted a RSVP target detection task (looking for a target, letter, color, or word among a series of distractors), while also memorizing the supra-threshold stimuli (images, pictures) that were consistently paired with the stimuli of the RSVP task. Different experiments conducted with this paradigm indicated that visual memory is enhanced for salient stimuli when paired with the targets of the RSVP task [13]–[16]. At first glance this result seems to contradict findings of slow-TIPL, where supra-threshold stimulus signals are detected, suppressed and not learned [11]. However, to date, fast-TIPL has been found only when subjects were explicitly asked to memorize the stimuli paired with the RSVP task [13]–[16]. When subjects were told to ignore the information presented with the RSVP stimuli, no TIPL was observed ([16] – Experiment 4, [17]). All together, the results obtained in fast-TIPL and slow-TIPL experiments show a benefit for salient stimuli that subjects process and no benefit for such stimuli that are ignored.

The objective of this paper is to determine the level of processing of the information paired with the RSVP task necessary to observe TIPL. More precisely, we want to study if attention to the irrelevant information paired with the RSVP task, but not an explicit memorization of them, is sufficient to observe TIPL. In order to answer this question, we conducted three experiments (Experiment 1A, Experiment 1B and Experiment 2). In the first experiment (1A), participants were asked to detect a target – a white square – in a stream of black and white squares while ignoring a second stream of images presented with the RSVP stimuli. The second experiment (1B) was identical except that participants were asked to memorize the images presented with the RSVP stimuli. The results of these first two experiments indicated that TIPL occurred when subjects were asked to memorize the images but not when they were asked to ignore them. To study if attention to the images without explicit memorization of them is sufficient to observe TIPL, Experiment 2 was conducted. As in previous experiments, participants were asked to detect a target in a stream of stimuli containing target and distractors, but they were also asked to detect a repetition in the image-stream. Results of Experiment 2 indicated TIPL, but interestingly only for male and not for female subjects. Indeed, analyses of data from a previous study of fast-TIPL demonstrate a consistent gender effect in fast-TIPL.

## Experiments 1A & 1B

### Methods

#### Participants

For Experiment 1A, of 24 participants, one was not included because of global performance inferior to 15% indicating that he did not perform the task, thus 23 participants were included (19 y.o.±17 months; 15 females, 8 males). For Experiment 1B, of 25 participants, two were not included because of a high level of false alarm (100%) indicating that they did not perform the task. Thus, 23 participants were included (20 y.o.±14 months; 14 females, 9 males). The participants gave written informed consent to participate in this experiment, which was approved by the Human Research Review Board of the University of California, Riverside. All participants reported normal or corrected-to-normal visual acuity and received course credit and financial compensation for the forty-minutes session.

#### Apparatus and Stimuli

An Apple Mac Mini running Matlab (Mathworks, Natick, MA) and Psychtoolbox Version 3 [18], [19] was used for stimulus generation and experiment control. Stimuli were presented on a 22″ CRT monitor with resolution of 1600×1200 and a refresh rate of 100 Hz. Participants sat with their eyes approximately 60 cm from the screen. The backgrounds of all displays were a mid-gray. Display items consisted of 108, 700×700 pixel (18.3 degrees of visual angle), photographs depicting natural or urban scenes from eight distinct categories (i.e., mountains, cityscapes, etc). Images were obtained from the LabelMe Natural and Urban Scenes database [20] at 250×250 pixels of resolution, then up-sampled to 700×700 pixels of resolution.

#### Procedure

**Figure 1:**
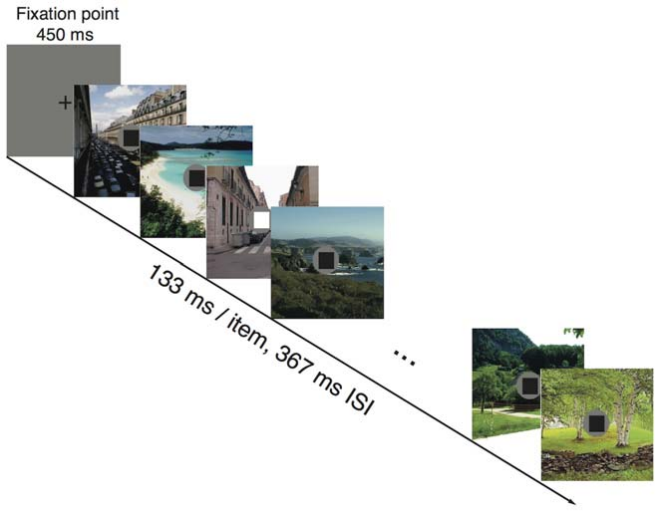
Design of Experiments 1A & 1B. Participants had to rapidly press the “Left Arrow” key when the white square appeared while also ignoring the images presented in RSVP (1A) or while also memorizing them (1B).

A stream of full-field images was presented in the middle of the screen. Each image was presented 133 ms, followed by a blank ISI of 367 ms for a SOA of 500 ms (Figure 1). For Experiment 1A, participants were informed that images will be presented but not to pay attention to them. For Experiment 1B, participants were told to memorize the images, and that an image recognition task would be performed at the end of the experiment.

For the Square Detection Task, a gray aperture (1 degree of visual angle and luminance of 92 cd/m^2^) was presented in the center of each image, thus centered in the middle of the screen. Each image was presented with a square (0.75 degree of visual angle) in the middle of the gray aperture. This square could be a distractor (black square; luminance of 0.25 cd/m^2^) or a target (white square; luminance of 251 cd/m^2^). Each square had the same onset and offset time as the image with which it was paired. The streams of stimuli (images and squares) were constructed with a trial format but without any perceptible break between trials. The image-stream consisted of 240 “trials”, with the presentation of 9 images per trial. In each trial, 1 image was paired with a white target square; the others 8 images were paired with black distractor squares. The white square target could appear in position 2 to 7. Thus, minimal interval between two targets was 3 images and the maximal interval was 13 images. The type of stimulus that an image coincided with (e.g. a target or a distractor) was held constant across the experiment. Of the 108 images, 12 images were paired with the target white square and the remained 96 images were paired with black square distractors with each image presented 20 times during the experiment. Image assignment to target and distractor was random for each participant. For both experiments, participants were asked to press the “LeftArrow” key as quickly as possible whenever they saw the white square and to make no response when a black square appeared. For each experiment, participants performed a practice block of 12 trials. Each participant was then tested for a total of 240 trials, in 10 blocks of 24 trials. Blocks were separated by brief breaks.

At the end of the experiment, participants of both experiments performed an Image Recognition Task. 48 images were presented to the participants: the 12 images paired with the target, 12 images paired with the distractor (randomly assigned for each participant) and 24 new images never presented in the experiment. One image was presented at a time until subjects made their response. For each image, participants were asked to report (by pressing the “UpArrow” or “DownArrow” keys) whether the test image had appeared during the Square Detection Task. The image recognition task was a surprise test for the participants of Experiment 1A but not for the participants of Experiment 1B.

For the analyses, paired t-tests and ANOVAs were primarily used. One-tailed tests were used to test our hypothesis that image recognition of target-paired items would be great than that for distractor-paired items. Two-tailed tests were used to test between other conditions.

### Results

Overall, mean performance on the white square detection task was 96.1%±1.1% (between-subject standard error) for Experiment 1A and 92.8%±1.7% for Experiment 1B. High performance on square detection indicated that participants complied with the instructions to maintain their gaze on the middle of the screen. A lack of significant differences between the experiments (t(22) = 0.22, p = .83) indicated that participants' memorization of images had little influence on performance of the central task.

**Figure 2:**
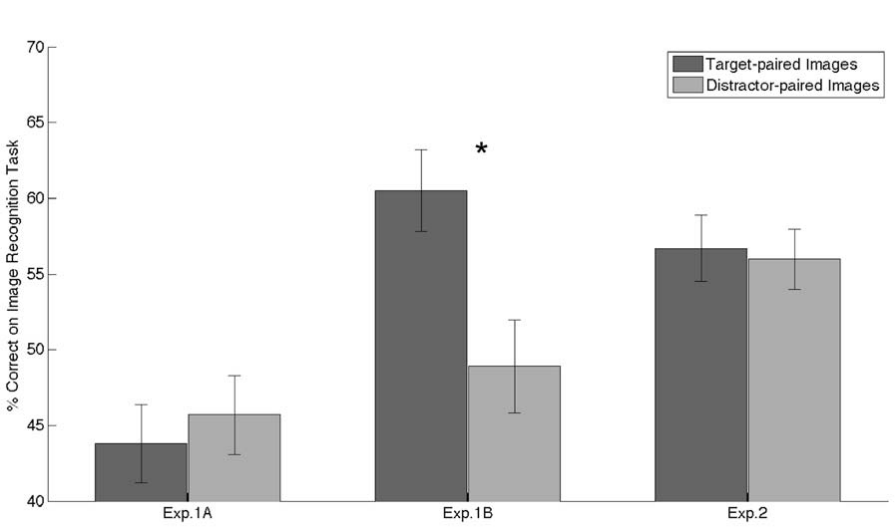
Results from the Image Recognition Task of Experiments 1A, 1B & 2. Plots represent accuracy (% correct). Error bars represent within standard error of the mean. Stars indicated significant comparisons.

Results for the image recognition task are shown in Figure 2. For Experiment 1A, hit rate for target-paired images (43.8%±2.6% (within-subject standard error)) and for distractor-paired images (45.7%±2.6%) were both larger than the false alarm (FA) rate (21.8%±2.8%), respectively t(22) = 4.63, p<.001 and t(22) = 6.84, p<.001. Also for Experiment 1B, hit rate for target-paired images (60.5%±2.7%) and for distractor-paired images (48.9%±3.1%) were both larger than the FA rate (17.0%±2.7%), respectively t(22) = 7.52, p<.001 and t(22) = 6.62, p<.001.

In order to study the existence of TIPL, we compared recognition performance between target-paired images and distractor-paired images in both experiments. For Experiment 1A, where subjects were not instructed to memorize the images, no significant difference was observed between recognition task accuracy (hits) for target-paired images vs. distractor-paired images, t(22) = −0.36, p = .64. Also, performance for target-paired images and distractor-paired images were not significantly different from chance, respectively t(22) = 1.57, p = .13 and t(22) = 1.10, p = .28. On the contrary, for Experiment 1B, where subjects were informed that there would be a memory test, significantly better recognition performance was obtained for target-paired images compared to distractor-paired images, t(22) = 2.11, p = .023. In this experiment hit rate for target-paired images was significantly larger than chance level, t(22) = 2.17, p = .041, however, performance for the distractor-paired images was not greater than chance, t(22) = 0.23, p = .82. The difference in TIPL between the both experiments was related to a difference in target-paired image recognition, t(45) = 2.67, p = .010, but not for distractor-paired image recognition, t(45) = 0.53, p = .60.

Results of Experiments 1A and 1B indicate fast-TIPL only when subjects were told to memorize the images, but not when they were told to ignore them. These findings are consistent with results of Swallow et al. [16], who found no differences in recognition between target-paired images and distractor-paired images when participants were not forewarned of the image recognition task. However, they do not substantiate those of Dewald et al. [17], who found that recognition for target-paired stimuli was worse than that of distractor-paired stimuli and than chance performance. While we found that performance in Experiment 1A was slightly worse for target-paired than distractor paired stimuli, this effect was not significant and both target and distractor paired recognition rates were greater than the false-positive rate. However, we were still interested in an inhibitory account of Dewald et al.'s [17] results, which could imply that the lack of TIPL could be due to an inhibition of the image stream. Such an account is similar to the finding of Tsushima, Seitz and Watanabe [10] who found inhibition in slow-TIPL, when salient stimuli were paired with task-targets. Given that we know that fast-TIPL can occur for salient target-paired images [13], it is clear that the level of processing of the images during the task mediates the acquisition of TIPL.

In order to study more deeply how processing of the images influences TIPL, we conducted Experiment 2 where we imposed a secondary task on the image-stream without asking subjects to memorize these images.

## Experiment 2

### Methods

#### Participants, Apparatus and Stimuli

Of 45 participants, three were not included; one because of global performance inferior to 10% and two because of a high level of false alarm (>80%) indicating that they did not perform the task, thus 42 participants were included (20 y.o.±2 y.o.; 21 females, 21 males). Participants were recruited and compensated in the same manner as in Experiments 1 and the stimuli were the same as described in Experiments 1, but 122 images were used in this experiment.

#### Procedure

This experiment was presented with a standard trial format, with 288 trials, where subjects reported after each trial whether one of the images in the image stream was presented twice. Each trial began with the presentation of a fixation cross for 450 ms. This presentation was followed by a rapid sequence of 9 full-field images. Each image was presented 133 ms, followed by a blank ISI of 367 ms for a SOA of 500 ms.

For the Square Detection Task, as in previous experiments, each image was presented with a black square (as distractor) or a white square (as target). The type of stimulus that an image coincided with (e.g. a target or distractor) was held constant across the experiment. Of the 122 images, 12 images were paired with the target white square and 96 images were paired with the black square distractor. Images paired with the target were presented 21 times each. Sixty of the images paired with distractors were presented 21 times each and the other 36 images were presented 22 times each (21 or 22 presentations were used to fit with the number of trials); only the images presented 21 times were employed in the memorization test. The number of repetitions was larger in this experiment than in Experiment 1 due to the addition of some trials in order to have enough data for the Repetition Image Task. Participants were asked to press the “Right Arrow” key as quickly as possible whenever they saw a white square (target) and to make no response when a black square (distractor) appeared.

For the Repetition Image Task, on half the trials (144 trials), one image was repeated, that is presented two times in rapid succession. Participants were instructed to detect in each trial if one image was repeated or not. At the end of each trial, when a blank screen appeared with the question “did you see an image repetition?” participants reported if they detected a repeated image (“Up Arrow” key), or not (“Down Arrow” key). The repeated image could be in position 1 to 8. When the repeated image was in position 1, for example, then the same image was presented in positions 1 and 2. Fourteen images of the 122 images were randomly assigned to be the repeated images. Eleven were presented 20 times (10 pairs) and 4 were presented 22 times (11 pairs). Of note, if the repeated image coincided with the target presentation, then the image associated with repetition was presented and not the image paired with the targets.

At the end of the experiment, participants performed an Image Recognition Task. 72 images were presented to the participants: the 12 images paired with the target, 12 images paired with the distractor (randomly assigned for each participant), 12 images used for the repeated images (randomly assigned for each participant) and 36 new images never presented in the experiment. One image was presented at a time and stayed on the screen until the subject made a response. For each image, participants were asked to report (by pressing the “UpArrow” or “DownArrow” keys) whether the test image had appeared in the experiment. This recognition task was a surprise to the participants.

### Results

Mean performance on the white square detection task was 93.2%±0.9% indicating that participants' detection of repeated images did not negatively influence performance of the central task. Overall, mean performance on the detection of image repetition was 80.8±1.4%.

Results for the image recognition task indicated that hit rate for target-paired images (56.7%±2.2%) and for distractor-paired images (56.0%±2.0%) were both larger than the FA rate (10.6%±1.2%), respectively t(41) = 15.85, p<.001 and t(41) = 15.95, p<.001. Also, hit rate for target-paired images was significantly larger than chance (t(41) = 2.11, p = .041), and a trend of significance was obtained for distractor-paired images (t(41) = 1.86, p = .070). However, no significant difference was observed between recognition task accuracy (hits) for target-paired images and distractor-paired images, t(41) = 0.20, p = .85 (Figure 2). Of note, participants' recognition for repeated-images was 79.4% (1.4%); the high performance for the repetition-paired images is not surprising, as the participants had to pay attention to these images in order to perform the Repetition Image Task.

**Figure 3:**
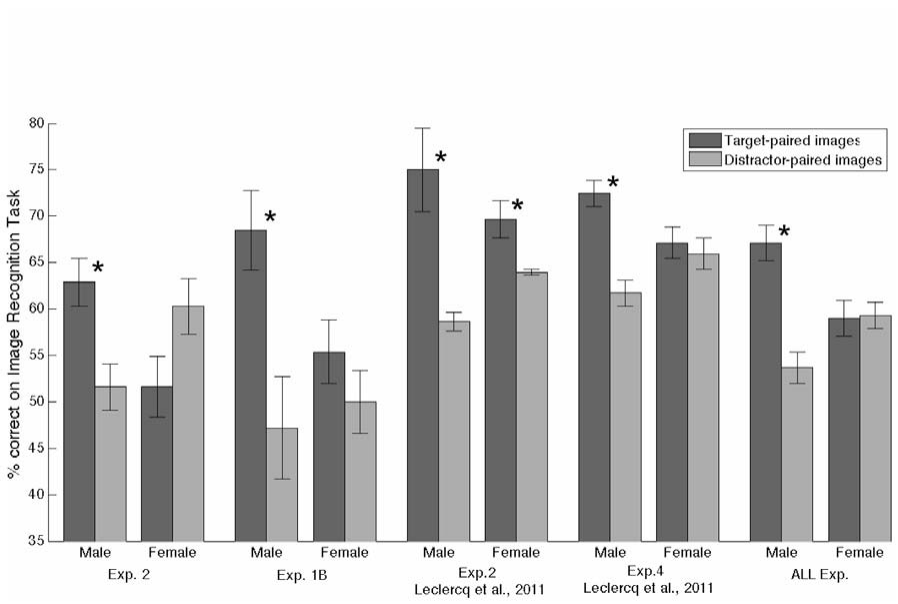
Gender Breakdown from the Image Recognition Task of Experiments in which TIPL was observed. Plots represent accuracy (% correct) from Experiments 1B & 2 of this paper and Experiments 2 and 4 of Leclercq and Seitz (2011) and the combined data from all of these experiments (plot named ‘ALL exp.’). Error bars represent within standard error of the mean. Stars indicated significant comparisons.

An interesting finding was observed when we examined fast-TIPL separately for male and for female subjects (Figure 3). An ANOVA with Pairing (Target; Distractor) as a within subjects factor and Gender (Male; Female) as a between subjects factor indicated a significant interaction between Pairing and Gender, F(1,40) = 6.37, p = .016. Planned t-tests showed a significant benefit for target-paired over distractor-paired image recognition for men, t(20) = 2.23, p = .038 (Target-paired = 61.9%±2.6% vs Distractor-paired = 51.6%±2.5%), but not for women, t(20) = 0.10, p = .92 (Target-paired = 51.6%±3.3% vs Distractor-paired = 60.3%±3.0%). Thus, fast-TIPL was found without the explicit instruction to memorize the images, but only in male subjects.

An important question raised by this result is whether this gender effect was a statistical fluke or whether it is generally valid. An analysis of the results obtained in Experiment 1B (Figure 3) also showed a significant difference between target-paired and distractor-paired images for men, t(8) = 2.90, p = .020 (Target-paired = 68.5%±4.3% vs Distractor-paired = 47.2%±5.5%), but not for women, t(13) = 1.28, p = .22 (55.4%±3.4% vs 50.0%±3.4%). To further evaluate gender differences in TIPL, we examined Experiments 2 and 4 from Leclercq & Seitz ([13]), where we had previously observed fast-TIPL. First, we combined the results of these two experiments with those of Experiments 1B and 2 of this paper and conducted an ANOVA on this dataset of 101 subjects (45 males and 56 females) (Figure 3) with Pairing (Target; Distractor) as within factor and Gender (Male; Female) as between factor. We found a significant effect of the Pairing, F(1,99) = 9.38, p = .003 and a significant interaction between the Pairing and Gender, F(1,99) = 10.49, p = .002, but no main effect of Gender, F(1,99) = 0.15, p = .70. Planned comparisons indicated better recognition performance for target-paired than distractor-paired images in men F(1,99) = 17.90, p<.001 (Target-paired = 67.1%±1.9% vs Distractor-paired = 53.7%±1.7%), but not in women F(1,99) = 0.02, p = .90 (Target-paired = 59.0%±1.9% vs Distractor-paired 59.3%±1.4%). Looking at the results of Leclercq & Seitz (2011) in more detail (Figure 3) we found a significant difference between target-paired and distractor-paired images for male subjects for both Experiment 2, t(5) = 2.73, p = .041 (Target-paired = 75.0%±4.5% vs Distractor-paired = 58.7%±1 0.%) and Experiment 4, t(8) = 4.31, p = .002 (72.5%±2.4% vs 61.7%±0.4%). However for female subjects, we found a significant effect in Experiment 2, t(13) = 2.80, p = .015 (Target-paired = 69.6%±2.0% vs Distractor-paired = 63.0%±0.3%) but not in Experiment 4, t(6) = 0.97, p = .37 (67.1%±2.9% vs 65.9%±0.4%). Overall, these differences in recognition performance between target-paired and distractor-paired images between men and women seemed related to a difference in the recognition performance on target-paired images, F(1,99) = 4.34, p = .040, and less to a difference on distractor-paired images, F(1,99) = 2.18, p = .14. These results supported a general difference in fast-TIPL between men and women, where TIPL is consistently found across studies in male subjects, but only inconsistently in female subjects.

**Figure 4:**
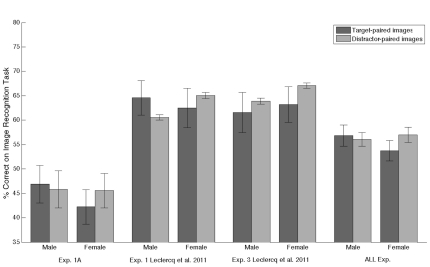
Gender Breakdown from the Image Recognition Task of Experiments in which TIPL was not observed. Plots represent accuracy (% correct) from Experiment 1A of this paper, Experiments 1 and 3 of Leclercq and Seitz (2011) and the combined data of all these experiments (plot named ‘ALL exp.’). Error bars represent within standard error of the mean.

Another question raised by this gender effect, concerned the existence of a gender effect in the experiments in which TIPL was not observed: Experiment 1A of this paper and Experiments 1 and 3 from our previous paper [13]. Figure 4, presents the results for each of these experiments and also the results for these experiments combined. An ANOVA conducted on the combined results of the 3 different experiments with Pairing (Target; Distractor) as within factor and Gender (Male; Female) as between factor revealed no effect of the factor Pairing (F(1,53) = 0.21, p = .65), nor interaction between the factors Pairing and Gender (F(1,53) = 0.65, p = .42). Looking at experiments individually, there were no significant fast-TIPL effect for men in Experiment 1A of this paper (Target-paired = 46.9%±3.8% vs Distractor-paired = 45.8%±3.8%; t(7) = 0.14, p = .90) nor in Experiment 1 (64.6%±3.5% vs 60.6%±0.5%; t(5) = 0.98, p = .37) or Experiment 3 (61.6%±4.1% vs 63.9%±0.6%; t(6) = −0.45, p = .67) of Leclercq & Seitz [13]. Likewise, there were no effects for women in Experiment 1A of this paper (Target-paired = 42.2%±3.5% vs Distractor-paired = 45.6%±3.5%; t(14) = −0.49, p = .64) nor in Experiment 1 (62.5%±4.1% vs 65.1%±0.6%; t(9) = −0.51, p = .62) or Experiment 3 (63.2%±3.7% vs 67.1%±0.6%; t(9) = −0.93, p = .38) of Leclercq & Seitz [13]. Thus, there is no gender effect in the experiments that did not produce TIPL; in other words, the absence of TIPL in these experiments cannot be explained by a gender effect.

In summary, results of Experiment 2 indicated that the process of the images without an explicit memorization of them is sufficient to observe fast-TIPL. However, this effect was observed only in male subjects. Further, we observe a general gender effect where fast-TIPL is consistently found in male but not female subjects. For female subjects, we often failed to observe fast-TIPL. However, these gender effects appeared to be specific to the TIPL effect in that no general performance differences were observed between men and women and no gender differences were found in experiments where fast-TIPL did not occur. Thus the absence of fast-TIPL in women cannot be attributed to a poor performance in women to memorize the images.

The repeated image task was designed as a method to require subjects to process the image stream without an explicit call to memorize the images, however, there is a possibility that some subjects used a memorization strategy, where they attempted to memorize each image and detected repetitions based upon images that they had seen before. Potential evidence for this is that the hit rate for the distractor-paired images in Experiment 2 (56.0%±2.0%) was larger than that in the Experiment 1B (48.9%±3.1%). However, this difference was non-significant (p = 0.21) and could be explained by the differences in subject group and by the increased processing of all the images in the stream in Experiment 2, even without explicit memorization of these stimuli. We also note that this would be a rather extreme strategy to adopt in our case given that in this experiment repetitions were immediate with just a 350 ISI between the images. Thus long term memory for this kind of task will be very consuming, and potentially problematic because it is normal for images to be repeated across trials and thus there would be a confusability in the long-term memory store between within and across trial image repetitions. Furthermore, we observed that memorization of images paired with repetition was better compared to images not paired with repetition. We suggest that this is because of the particular attention that subjects pay to those images when they were repeated in order to correctly answer to the repetition task.

## Discussion

Our results showed three main findings. First, that fast-TIPL occurred when subjects were instructed to attend to memorize the image-stream but not when they were told to ignore the image-stream. Second, that when subjects were required to attend to the image-stream but not instructed to memorize them, fast-TIPL was found, but only for male subjects. Lastly, looking across multiple studies of fast-TIPL, we found that this gender effect was robust, where male subjects consistently show fast-TIPL, whereas the observation of fast-TIPL is inconsistent in female subjects. We discuss each of these results below.

Previous experiments on fast-TIPL indicated fast-TIPL when subjects are asked to memorize the images, but not when they are asked to ignore them [16], [17]. The results obtained in Experiments 1A, 1B corroborate the finding that fast-TIPL is not obtained when subjects ignore the image stream during training [16]. However, we failed to replicate Dewald et al.'s [17] finding of inhibition for the target-paired images compared to the distractor-paired images. It is possible that these disparate results could be due to the different frequencies with which items in the irrelevant image-stream were presented. In Dewald et al.'s experiment [17], each irrelevant image was presented only 2 times, whereas in Swallow et al. [16] each irrelevant image was presented 10 times, and in Experiments 1A and 1B, of this paper, each image was presented 20 times. A greater frequency of presentation of the target-paired images in our experiments and in that of Swallow et al. [16] could account for why inhibition was not observed. It is possible that increase the frequency of presentation of the irrelevant information allows the system to memorize this information even if participants do not pay attention (at least explicitly) to this information. However, our first data indicate equivalent results compared to Swallow et al. [16] even with the use of a higher frequency. More experiments are necessary to study more deeply the frequency effect.

Experiment 2 was conducted to study if processing the images in a secondary task, but without a memorization requirement, was sufficient to obtain fast-TIPL. The results indicate that even if participants were not told to memorize the images, and these images were not the targets of the image repetition task, target-paired images were memorized at a higher rate than distractor paired-images (although as discussed below, this finding was only true for the male subjects in the study). Thus, the processing of the images without explicit memorization is sufficient for target-pairing to benefit to the later recall of those images. These results are highly consistent with previous findings of TIPL: inhibition of target-paired stimuli is found for salient, irrelevant, and distracting stimuli [10], [11], but facilitation is found for stimuli that are relevant to another task, although still irrelevant for the main RSVP task that the subjects are conducting [21]. This observation is consistent with recent models of TIPL [5], [9] that suggest that attentional signals can either enhance or suppress Perceptual Learning (both task-relevant and task-irrelevant) depending on whether those stimuli are distracting or not to the subjects' objectives.

An important result revealed by our experiments is the existence of a gender effect in fast-TIPL. An analysis of the results of different experiments conduct in our laboratory indicated that this gender effect is in fact consistent across multiple studies, where fast-TIPL occurs consistently in male but not in female subjects. Notably, no other gender effect was observed in our analyses, and the equivalent performance on recognition for the repeated images in Experiment 2 between female (79.8) and male (79.0), indicated that the absence of fast-TIPL in women wasn't attributable to overall performance differences in women compared to men. As such it appears that the gender difference is directly related to the phenomenon of fast-TIPL.

Without further research on the topic we can only speculate regarding the source of this gender difference. Different neuromodulatory systems in the brain have been proposed to have a role in learning [3], [22]. The norepinephrine system has an important role in learning, notably by synaptic plasticity [23], [24]; the acetylcholine neuromodulatory system has been shown to modulate perceptual learning [25], [26] and cortical plasticity [27]–[29]. Finally, the dopamine system plays important roles in learning and plasticity [30], [22]. One possibility is that the difference in fast-TIPL between male and female subjects could be related to a difference in the release of a neuromodulator at the relevant point in time (detection of the target); for example some studies suggest greater dopamine release in men compared to women [31]. Another possibility relates to gender differences in spatial abilities, where there is evidence of a difference in global/local bias with a local bias for women and a global bias for men (see [32]). A local bias to attend the target when it appears would reduce the occurrence of fast-TIPL for target-paired images in women compared to men. It may also be the case that the images used in our experiment were processed differently by men and by women. The images that we employed were mostly landscapes that could require a more global process. If this is true, then a different image set could lead to greater fast-TIPL in women than in men. However, to date, examinations of gender differences in global–local processing are sparse, and the results are inconsistent [32]. Further research will be required to address these possibilities and to better understand the source of the gender effect.

In conclusion, our results indicated that the level of processing of target-paired information influences fast-TIPL. Processing this information without an explicit memorization is sufficient for TIPL to occur. However, looking across multiple studies of fast-TIPL, we found a robust gender effect, where male subjects consistently show fast-TIPL, whereas the observation of fast-TIPL is inconsistent in female subjects.
